# Intensive Home Care for COVID-19 to Reduce Admissions

**DOI:** 10.1177/10848223211035718

**Published:** 2021-11

**Authors:** Rachel Aviv, Madeline Abrams, Fiore Mastroianni, Marcia Epstein, Gita Lisker

**Affiliations:** 1 Division of Pulmonary, Critical Care, and Sleep Medicine, Department of Medicine, Zucker School of Medicine, New Hyde Park, NY, USA; 2 Donald and Barbara Zucker School of Medicine at Hofstra/Northwell, Hempstead, NY, USA; 3 Division of Infectious Diseases, Department of Medicine, Zucker School of Medicine, Manhasset, NY, USA

**Keywords:** COVID-19, home care services, hospitalization, telemedicine, delivery of health care, antiviral agents

## Abstract

Hospitalization for COVID-19 has placed a significant financial and logistical burden on hospitals and health care systems. Limitations on visitation and isolation precautions have made hospitalization more isolating for patients in the time of COVID-19. Increasing the provision of healthcare delivered at home has the potential to decrease healthcare costs by providing care at home which may be preferred for many patients. We describe a series of 39 patients who were treated with intravenous remdesivir at home in addition to oxygen, dexamethasone, and anticoagulants. These patients were at high risk for decompensation due to COVID-19 and met accepted criteria for admission—need for supplemental oxygen and intravenous remdesivir. All patients had home lab monitoring and frequent telehealth visits. Over the study period 13 (33%) of patients were admitted for worsening COVID-19 and 5 (13%) died. Twenty-six patients avoided admission, and none experienced a severe adverse effect from in-home treatment. The expanded use of telehealth services due to the COVID-19 pandemic has the potential to increase the frequency of patient monitoring by physicians and the provision of care and monitoring usually restricted to hospitalized patients.

## Introduction

Hospitalization for respiratory failure due to COVID-19 is frequent, however several treatments for COVID-19 can be delivered in the home obviating the need for hospitalization. In-hospital treatments for COVID-19 include dexamethasone, remdesivir, oxygen, and prophylactic anticoagulation. Laboratory monitoring is frequent during hospitalization and can be used to detect adverse events of drugs and clinical deterioration. Oral dexamethasone and oxygen are commonly prescribed to outpatients, however daily administration of remdesivir has not been reported. The need for IV access, oxygen, regular monitoring by physicians and nursing staff drives many admissions for COVID-19.

While remdesivir is currently approved for patients hospitalized with COVID-19, any benefit from remdesivir use is likely to be early in the disease course.^1^ We conducted a retrospective analysis of outpatient use of intravenous remdesivir to examine the effect of providing intensive home care on the need for admission in ill outpatients who otherwise met criteria for admission. All patients had been enrolled in our health system’s outpatient COVID-19 treatment program which consists of telehealth visits with physicians and nurse practitioners, in-home laboratory testing, imaging, and in-home infusions of intravenous fluids and other medications.^2^

Beginning in December 2020, remdesivir was offered in addition to standard care to patients requiring in-home supplemental oxygen and deemed moderate to severe by the treating physician. Informed consent was obtained prior to administration of remdesivir. An unrelated trial of intravenous remdesivir in outpatients with mid COVID-19, sponsored by Gilead Sciences, was recently stopped (NCT04501952).^3^

## Methods

We conducted a retrospective review of all cases of outpatient remdesivir use through our outpatient COVID-19 treatment program between December 2020 and March 2021 as these patients would have otherwise required admission to receive the drug. The primary study endpoints were hospital admission for COVID-19 and death from any cause at 28 days. We recorded adverse events including elevation in serum creatinine or liver tests, IV infiltration, and allergic reaction to remdesivir.

Continuous variables are reported as mean ± standard deviation (SD) or median, interquartile range (IQR), as appropriate. Comparisons were made using a 2-tailed Wilcoxon Signed-Rank test. The study was approved by the Northwell Health Institutional Review Board and informed consent was waived.

## Results

A total of 39 patients received remdesivir during the study period. The mean age was 62.3 ± 13.2 years and 21 (54%) were men. Patients received a median of 5 (IQR: 2-5) doses of remdesivir and began treatment 7.4 ± 3.8 days after testing positive for COVID-19. Thirty-three (85%) patients were treated with glucocorticoids, 29 (74%) received oxygen, 14 (36%) were treated with prophylactic dose oral anticoagulant, and 10 (26%) received an anti-SARS-CoV-2 monoclonal antibody (Table 1). At 28 days post-treatment initiation, 13 (33%) patients had been hospitalized, all for worsening respiratory failure. No patient was admitted for a drug-related adverse effect.

**Table 1.** Demographic Data for All Patients Treated with Remdesivir.

	Total (n = 39)
Age years (mean ± SD)	62.3 ± 13.2
Male, n (%)	21 (54)
Race, n (%)	
Asian	4 (10)
Black	3 (8)
White	26 (67)
Other/unknown	6 (15)
Comorbidities, n (%)	
Obesity (BMI > 30 kg/m^2^)	8 (21)
Hypertension	17 (44)
Diabetes	5 (13)
Any comorbidity	21 (54)
Time from diagnosis to treatment, days, mean (SD)	7.4 ± 3.8
Total remdesivir doses, median (IQR)	5 (2-5)
Other therapies, n (%)	
Oxygen	29 (74)
Glucocorticoids*	33 (85)
Monoclonal antibody	10 (26)
Prophylactic anticoagulation^†^	14 (36)

* Glucocorticoids include both dexamethasone and prednisone.

† Prophylactic anticoagulation includes both rivaroxaban and apixaban.

Five patients (13%) in this cohort died during the study period and all 5 deaths were due to respiratory failure from COVID-19 or complications of COVID-19. Three of these patients had been referred to hospital by the physician during their initial visit but declined, and all 3 presented to emergency rooms the following day after receiving 1 or 2 doses of remdesivir. One patient who had not been initially recommended hospitalization also received 1 dose of remdesivir at home before presenting to hospital and 1 patient had worsening respiratory failure 1 week after completion of remdesivir, ultimately choosing hospice care.

All patients had an initial telehealth visit with a physician or nurse practitioner and at least 1 telehealth visit during their treatment course to monitor for adverse effects, improvement, or decompensation. All patients had either a telehealth visit, or in-person visit after their treatment course with remdesivir.

Lab tests were recorded from results immediately before drug administration and on the last day of therapy. There was a significant decrease in creatinine (0.93-0.89 mg/dL, *p* = .006). There were no significant differences in the AST (35-25 U/L, *p* = .06), ALT (29-36 U/L, *p* = .07), or total bilirubin (0.4-0.5 mg/dL, *p* = .05). One patient (3%) had an elevation of serum creatinine of >0.3 mg/dL over the study period (absolute increase 0.37 mg/dL) and 1 patient (3%) had elevation of ALT to 5 times the upper limit of normal. There were no significant abnormalities of serum AST or total bilirubin (Table 2).

**Table 2.** Primary and Secondary Outcomes.

	Total (n = 39)		
Outcomes, n (%)			
Hospital admission	13 (33)		
Death	5 (13)		
IV infiltration	0 (0)		
Allergic reaction	0 (0)		
Adverse effects, n (%)			
Creatinine increase >0.3 mg/dL	1 (3)		
AST >2× ULN	1 (3)		
ALT >2× ULN	6 (15)		
Total bilirubin >2× ULN	0		
Baseline lab values, median (IQR)	Before (n = 36)	After (n = 36)	*p*-value*
Creatinine (mg/dL)	0.93 (0.85-1.05)	0.89 (0.77-0.97)	.006
AST (U/L)	35 (29-40)	25 (22-42)	.06
ALT (U/L)	29 (24-48)	36 (26-56)	.07
Total bilirubin (mg/dL)	0.4 (0.3-0.5)	0.5 (0.3-0.6)	.05

Lab data was only available for 36 of 39 patients in the cohort. Before lab values were recorded from blood drawn on the first day of remdesivir. After values were those recorded on the last day of therapy.

* Reported for Wilcoxon Signed-Rank test.

No patients had IV infiltration or an allergic reaction to remdesivir.

## Discussion

All study patients could have been admitted for COVID-19 pneumonia with hypoxemia and treatment with intravenous remdesivir. Remdesivir was administered at home to patients who required supplemental oxygen or were considered high risk for complications from COVID-19 with few adverse effects. There were no clinically significant changes in creatinine or liver enzymes over the course of drug administration. One patient had a significant increase in ALT over the course of treatment but had no change in AST or total bilirubin.

Thirteen percent of patients died, a fraction similar to previous studies describing inpatient mortality due to COVID-19 in the United States.^4,5^ The majority (3/5) of deaths occurred in patients who were initially deemed sick enough to warrant referral to the hospital, however were offered home management after they declined hospitalization. These patients were ultimately hospitalized the following day after receiving only 1 or 2 doses of remdesivir. Intensive at-home monitoring via telehealth and incorporating daily nurse visits to administer drugs and draw blood for labs can reduce the need for admission and may even serve to enforce the need for hospitalization in sick patients.

Limitations of this study include its retrospective, observational design, non-random allocation of remdesivir, small sample size, and lack of control group. The outcomes should drive future design of studies and systems. Frequent monitoring and nursing care can be delivered in the home with regular intravenous access and blood draws.

With close follow up, drug adverse events and administration complications can be avoided or mitigated. The low rate of adverse effects is encouraging and should prompt continued investigation into the at-home use of remdesivir and other interventions for the treatment of COVID-19.

Future scaling and development of similar integrated home healthcare systems may be beneficial to patients and may reduce overall healthcare costs without compromising quality of care.^6^ Treatments like prolonged infusions of antimicrobials and other drugs could similarly be administered in the home setting with close monitoring and laboratory follow up.
